# Inhibitory effect and mechanism of gelatin stabilized ferrous sulfide nanoparticles on porcine reproductive and respiratory syndrome virus

**DOI:** 10.1186/s12951-022-01281-4

**Published:** 2022-02-05

**Authors:** Ting Tong, Shuangfei Deng, Xiaotong Zhang, Liurong Fang, Jiangong Liang, Shaobo Xiao

**Affiliations:** 1 College of Resource and Environment, College of Science, State Key Laboratory of Agricultural Microbiology, Huazhong Agricultural University, No.1 Shizishan Street, Hongshan District, Wuhan, 430070 Hubei People’s Republic of China; 2 State Key Laboratory of Agricultural Microbiology, College of Veterinary Medicine, Key Laboratory of Preventive Veterinary Medicine in Hubei Province, The Cooperative Innovation Center for Sustainable Pig Production, Huazhong Agricultural University, No.1 Shizishan Street, Hongshan District, Wuhan, 430070 Hubei People’s Republic of China

**Keywords:** Ferrous sulfide nanoparticles, Antiviral mechanism, Porcine reproductive and respiratory syndrome virus, Inactivation, Adsorption, Invasion, Replication, Release

## Abstract

**Background:**

The infection and spread of porcine reproductive and respiratory syndrome virus (PRRSV) pose a serious threat to the global pig industry, and inhibiting the viral infection process is a promising treatment strategy. Nanomaterials can interact with viruses and have attracted much attention due to their large specific surface area and unique physicochemical properties. Ferrous sulfide nanoparticles (FeS NPs) with the characteristics of high reactivity, large specific surface area, and low cost are widely applied to environmental remediation, catalysis, energy storage and medicine. However, there is no report on the application of FeS NPs in the antiviral field. In this study, gelatin stabilized FeS nanoparticles (Gel-FeS NPs) were large-scale synthesized rapidly by the one-pot method of co-precipitation of Fe^2+^ and S^2‒^.

**Results:**

The prepared Gel-FeS NPs exhibited good stability and dispersibility with an average diameter of 47.3 nm. Additionally, they were characterized with good biocompatibility and high antiviral activity against PRRSV proliferation in the stages of adsorption, invasion, and replication.

**Conclusions:**

We reported for the first time the virucidal and antiviral activity of Gel-FeS NPs. The synthesized Gel-FeS NPs exhibited good dispersibility and biocompatibility as well as effective inhibition on PRRSV proliferation. Moreover, the Fe^2+^ released from degraded Gel-FeS NPs still displayed an antiviral effect, demonstrating the advantage of Gel-FeS NPs as an antiviral nanomaterial compared to other nanomaterials. This work highlighted the antiviral effect of Gel-FeS NPs and provided a new strategy for ferrous-based nanoparticles against PRRSV.

**Graphical Abstract:**

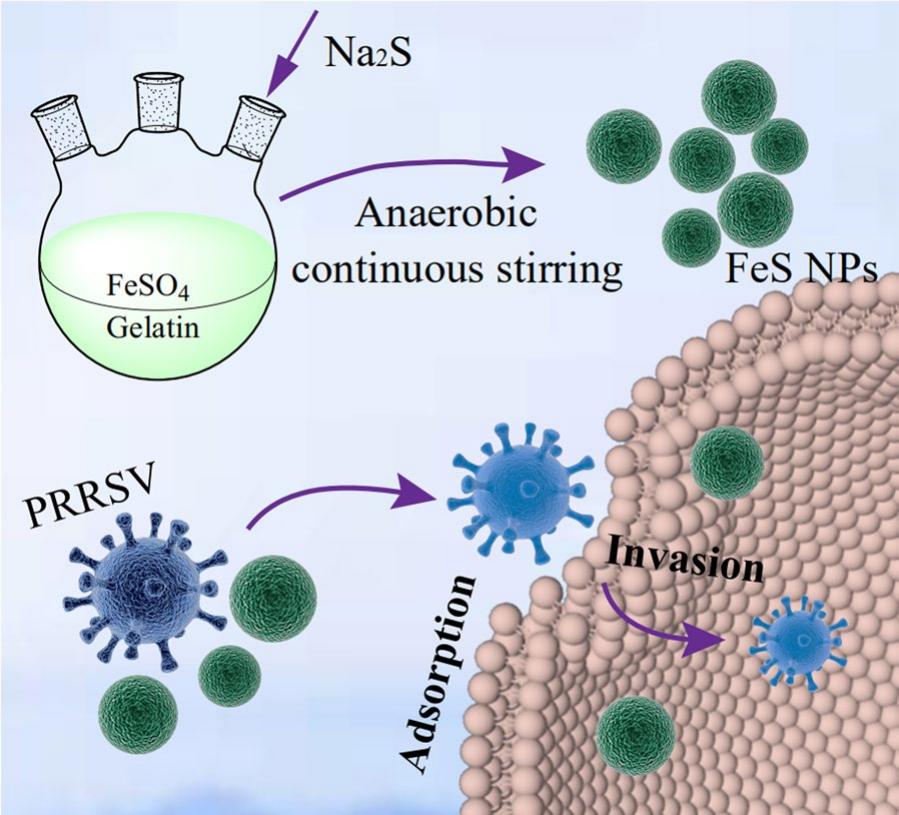

**Supplementary Information:**

The online version contains supplementary material available at 10.1186/s12951-022-01281-4.

## Introduction

Porcine reproductive and respiratory syndrome (PRRS) is a disease that causes reproductive failure in breeding sows and respiratory disorders in growing pigs [1]. The etiologic agent, PRRS virus (PRRSV), an enveloped, single positive-stranded RNA virus of the family *Arteriviridae* [2], has caused huge economic losses in the swine industry worldwide in the past three decades, due to its persistent mutation and epidemic caused by the high variability of the RNA virus genome, making its prevention and treatment still unsatisfactory [3]. This suggests the necessity to develop novel antiviral strategies against PRRSV by inactivating the virus from the initial stage of infection.

In recent years, a series of innovative nanomaterials with potential activities against various viruses have been reported to play an important role in preventing and treating different viral infections [4–8], such as DNA origami [9], graphene nanosheets [10, 11], fullerene nanospheres [12–14], macromolecular polymers [15], nano hydrogels [16–18], and other emerging materials [19, 20]. These nanomaterials can play an antiviral role in the different stages of the virus life cycle, but their further application was restricted by the low synthesis yield and the complex synthesis procedures, suggesting the rare advantage of the one-pot method for rapid and large-scale synthesis of antiviral nanomaterials with good reproducibility.

Meanwhile, iron-based antiviral materials have also attracted much attention. Iron ions are essential trace elements for cell growth and prevention of cancer, cardiovascular disease, and diabetes [21, 22]. The iron-based complexes were reported to have a specific antiviral effect on various viruses [23, 24], and the use of iron ions as a supplementary therapy proved to be a promising antiviral strategy [25, 26]. However, the strong oxidizability of iron ions may have a certain degree of harm to cells, despite the extensive application of iron supplement with ferrous ions in the prevention and treatment of iron deficiency anemia [27, 28]. Owing to its specific physical and chemical properties, ferrous sulfide nanoparticles (FeS NPs) has been studied in many fields, such as anti-tumor, energy, sewage treatment, and so on [29, 30], but to our knowledge, no study has ever been performed on its antiviral effects. Due to the instability and easy oxidation of FeS in aqueous solution, we improved the stability of FeS NPs by introducing gelatin into the reaction system [31]. As a biomedical material, gelatin has various attractive features, such as biocompatibility, low immunogenicity, biodegradability, and easy manipulation [32, 33].

In this study, we reported the inhibitory effect and mechanism of gelatin stabilized ferrous sulfide nanoparticles (Gel-FeS NPs) on PRRSV. The antiviral agent was synthesized by the co-precipitation method, and Gel-FeS NPs were shown to possess strong inhibitory effects against PRRSV proliferation, not only inactivating the PRRS virions, but also suppressing PRRSV adsorption, invasion, and replication in the host cell. To the best of our knowledge, this is the first report about the virucidal and antiviral activity of ferrous-based nanoparticles. This work highlighted the antiviral effect of Gel-FeS NPs and provided a new strategy for ferrous-based nanoparticles against PRRSV.

## Results

### Characterization of Gel-FeS NPs

**Fig. 1:**
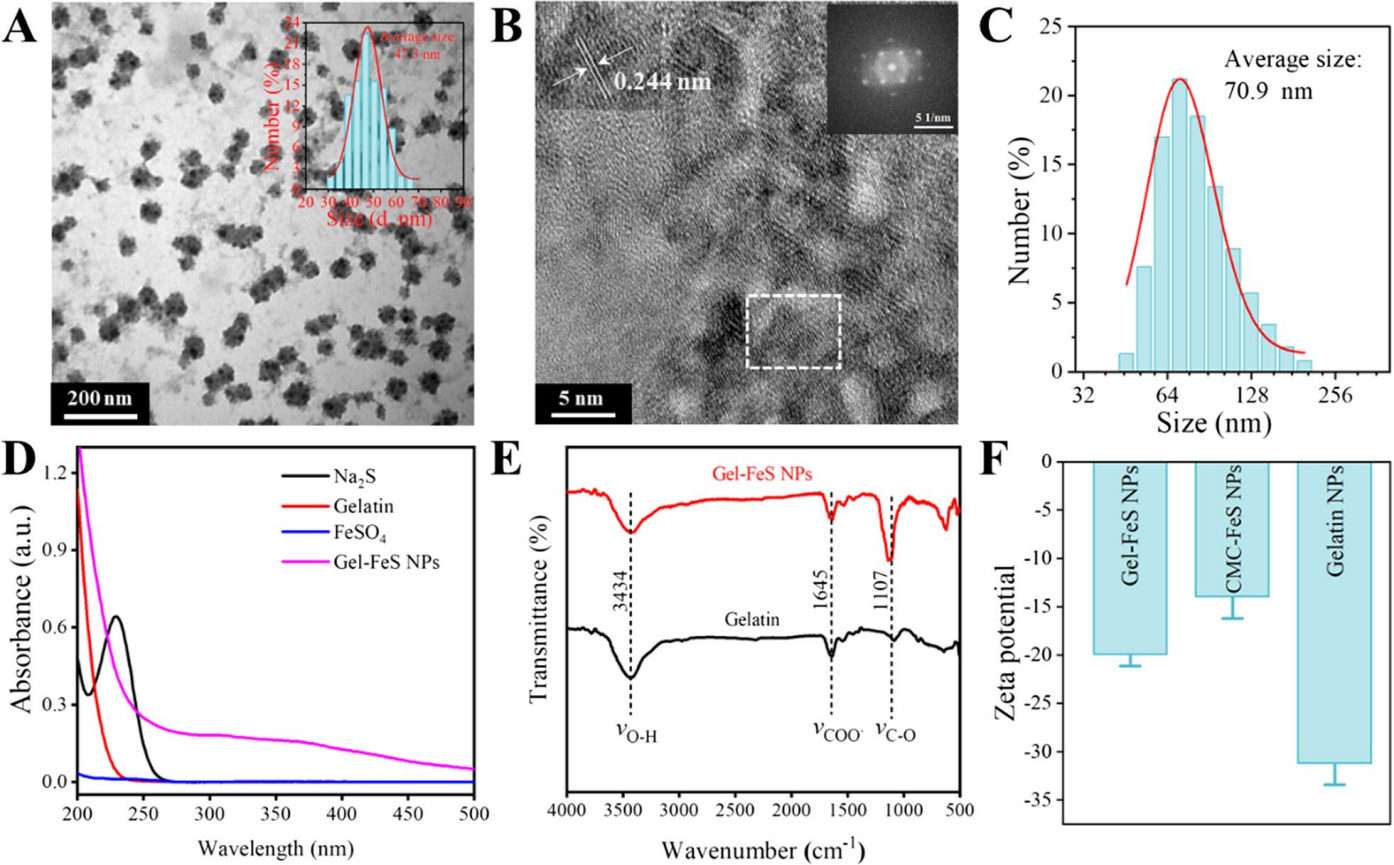
Characterization of Gel-FeS NPs. **A** TEM image of the Gel-FeS NPs, with the inset for the size distribution histograms of corresponding Gel-FeS NPs. **B** HR-TEM images of Gel-FeS NPs, with the inset for lattice diffraction pattern of corresponding Gel-FeS NPs. **C** DLS analysis of Gel-FeS NPs. **D** UV–Vis spectra of Gel-FeS NPs, FeSO_4_, Na_2_S, and gelatin. **E** FTIR spectra of Gel-FeS NPs, and gelatin. **F** Zeta potential distribution of Gel-FeS NPs, CMC-FeS NPs, and Gelatin NPs

Gelatin stabilized FeS NPs were simply synthesized by the co-precipitation method [34]. The precursors containing the ferrous ions of FeSO_4_ and S^2−^ of Na_2_S were co-precipitated in the nitrogen-protected aqueous solution under continuous stirring. The morphology and size distribution of Gel-FeS NPs were measured using a transmission electron microscope (TEM) and dynamic light scattering (DLS) analyzer. In the TEM image (Fig. 1A), Gel-FeS NPs were seen to be well-dispersed in aqueous solution with a uniform size, and 100 nanoparticles were selected to calculate the diameter of Gel-FeS NPs, which showed an average diameter of 47.3 nm. Moreover, the lattice structure in the interior of Gel-FeS NPs can be observed in the high-resolution TEM images (Fig. 1B), and Gel-FeS NPs presented a clear lattice diffraction pattern with a lattice spacing of 0.244 nm. The average diameter of hydrated particles of Gel-FeS NPs was measured to be 70.9 nm by dynamic light scattering (DLS) instrument (Fig. 1C). Figure 1D shows the UV–Vis spectra of FeSO_4_, Na_2_S, gelatin, and Gel-FeS NPs, with negligible absorption for the individual solutions of FeSO_4_, Na_2_S and gelatin in the longer wavelength region over 250 nm. After mixing these three reagents and reaction, the black product could be observed, which showed strong absorbance in the wavelength range of 200–500 nm, further confirming the successful formation of Gel-FeS NPs. After storage at 4 °C for a week, Gel-FeS NPs showed no notable change in the UV–Vis spectra (Additional file 1: Fig. S1), implying their good physical and chemical stability in different aqueous solutions (Additional file 1: Fig. S2).

The molecular structure and chemical bond information were obtained by Fourier transmission infrared spectroscopy (FTIR) analysis of Gel-FeS NPs and gelatin to study the differences in functional groups (Fig. 1E). The peaks at 3434 cm^−1^ were assigned to the O−H stretching vibrations from adsorbed water and the stabilizer gelation. The abundant O−H bonds in gelatin were attributed to the formation of strong intermolecular hydrogen bonds between gelatin and FeS NPs, which improved the stability of Gel-FeS NPs effectively. Moreover, the absorption peaks at around 1645 cm^−1^ and 1107 cm^−1^ indicated the stretching vibrations of −COO^−^ and C−O−C, respectively. The modification of gelatin provided electrostatic repulsion and steric hindrance for FeS to avoid oxidation and aggregation [35]. In order to compare the properties of Gel-FeS NPs, carboxymethyl cellulose (CMC) modified FeS nanoparticles (CMC-FeS NPs) were synthesized using the one-pot method of co-precipitation of Fe^2+^ and S^2‒^, gelatin nanoparticles (Gelatin NPs) were synthesized by the crosslinking method, the former of which is elliptical, while the latter is circular (Additional file 1: Fig. S3). Dynamic light scattering results showed that their hydrated particle sizes are 95.0 nm and 148.4 nm, respectively (Additional file 1: Fig. S4). Zeta potentials of the three nanoparticles were recorded in Fig. 1F.

**Fig. 2:**
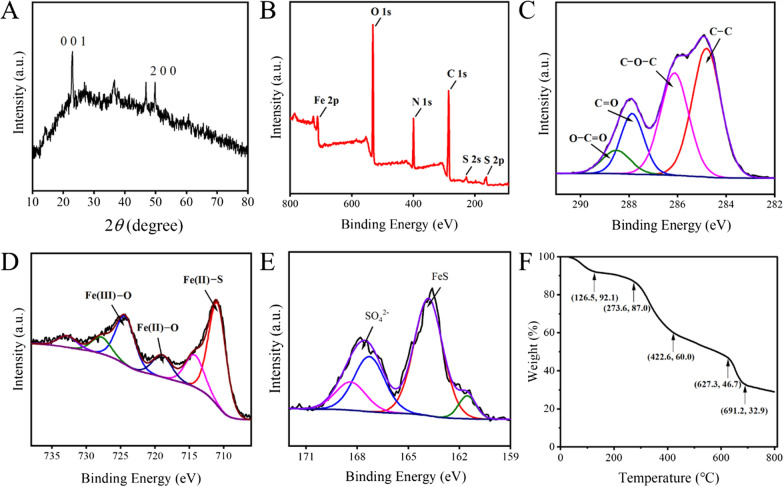
**A** XRD pattern of Gel-FeS NPs. **B** X-ray photoelectron spectroscopy (XPS) full scans spectrum of Gel-FeS NPs and high-resolution XPS spectra of **C** C 1 s, **D** Fe 2p, **E** S 2p. **F** Thermogravimetric analysis (TGA) of Gel-FeS NPs with the percentage weight loss of the sample as a function of temperature

Meanwhile, the XRD diffraction pattern of Gel-FeS NPs was analyzed (Fig. 2A). The two typical peaks at 2*θ* = 23.0° and 47.0° represented FeS, and the diffraction peak at 2*θ* = 49.8° corresponded to (2 0 0) reflection of FeS, indicating the crystal form of FeS in the presence of gelatin [36]. Additionally, the peak at 2*θ* = 36.4° corresponded to iron oxides, probably due to partial oxidation of Gel-FeS NPs during sample drying and analysis. In Fig. 2B, the components and surface functional groups of Gel-FeS NPs were characterized by X-ray photoelectron spectroscopy (XPS) analysis, and the XPS full scan spectrum exhibited five obvious peaks at the binding energy of 163.30, 399.14, 530.98, 582.20 and 710.57 eV, corresponding to S 2p, C 1 s, N 1 s, O 1 s, and Fe 2p orbital, respectively. The high resolution XPS spectra of C 1 s could be resolved into four peaks at 284.81, 286.12, 287.87, and 288.52 eV, indicating the presence of C−C, C−O−C, C=O, and O−C=O bonds in Gel-FeS NPs, respectively (Fig. 2C). In the high resolution XPS spectra of Fe 2p (Fig. 2D), the peak at 710.92 eV was assigned to Fe(II)−S species, the main form of element Fe in Gel-FeS NPs, and the peaks at 719.04 and 724.33 eV were attributed to Fe(II)−O and Fe(III)−O, respectively, implying the partial oxidization of Gel-FeS NPs during storage and detection as previously reported [37]. In the high resolution XPS spectra of S 2p (Fig. 2E), the peak at 161.50 eV was ascribed to FeS, corresponding to the peaks of Fe 2p at 710.92 eV, and the two peaks at 167.41 and 168.30 eV of SO_4_^2−^ indicated that some sulphate impurities could not be removed thoroughly.

Gelatin is a biocompatible material without obvious biological toxicity, and we did the thermogravimetric analysis (TGA) of Gel-FeS NPs to calculate the loading of gelatin (Fig. 2F). The total mass loss for Gel-FeS NPs was seen to reach 72% at 800 ℃, and the process of mass loss under heating can be tentatively divided into several stages: (I) 0–273.6 ℃, (II) 273.6–422.6 ℃, (III) 422.6–691.2℃, and (IV) above 691.2 ℃. According to previous reports [34, 38], water was the only product for Gel-FeS NPs heated up to 126.5 ℃, while the temperature up to 273.6 ℃ (stage I) corresponds to the release of water and the loss of loosely attached gelatin molecules in the absence of thermal degradation of Gel-FeS NPs. Stage II (273.6–422.6 ℃) and stage III (422.6–691.2 ℃) are associated with gelatin degradation and thermal decomposition of gelatin on the surface of modified nanoparticles. After reaching 691.2 ℃, ferrous sulfide remained. Based on the TGA results, the content of FeS NPs in the whole Gel-FeS NPs is about 28%, and further calculation indicated that the content of iron is 17.8%.

### Biocompatibility of Gel-FeS NPs

**Fig. 3:**
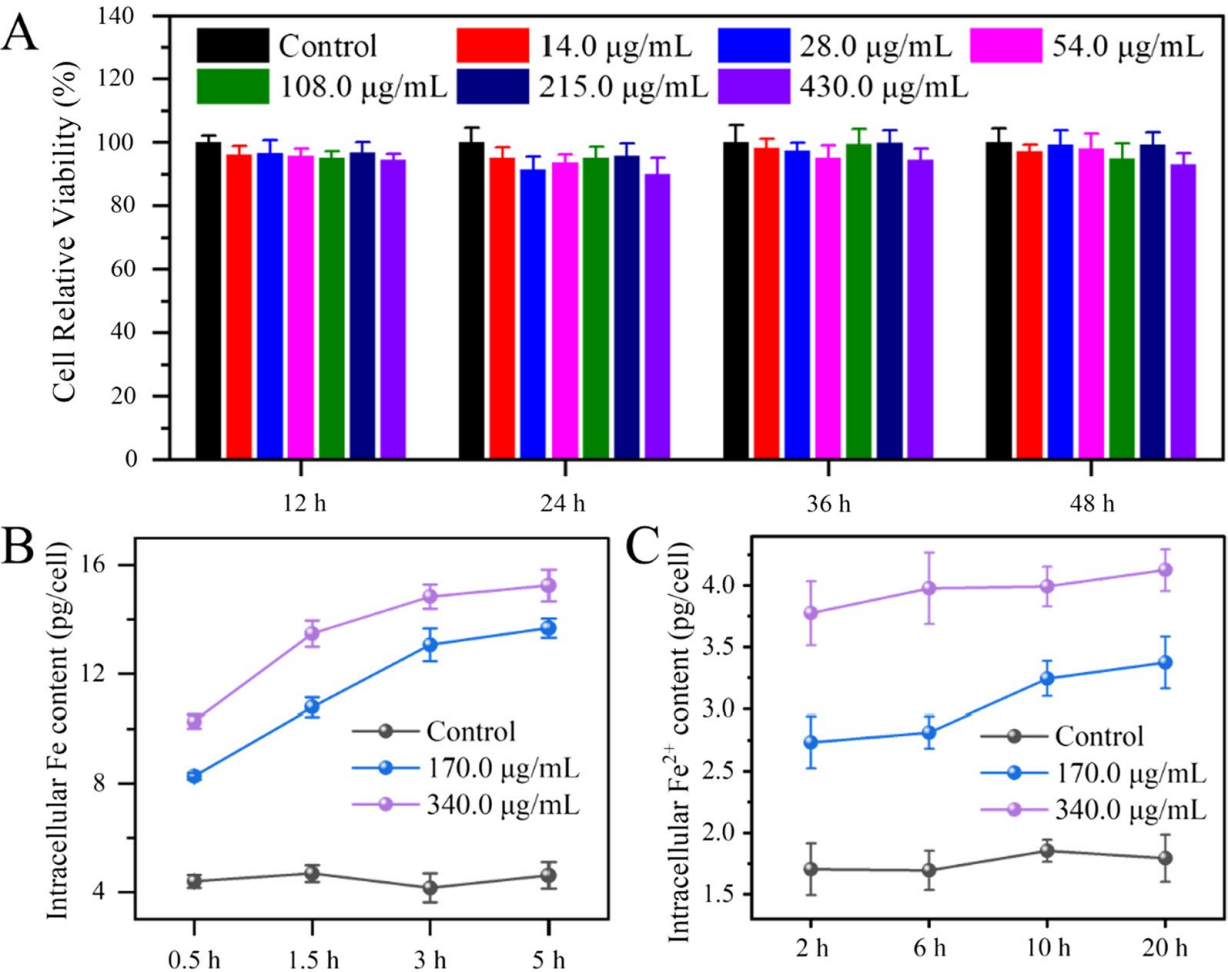
**A** Cell cytotoxicity of different concentrations of Gel-FeS NPs (0‒430.0 µg/mL) on MARC-145 cells. **B** The cellular internalization experiment of Gel-FeS NPs. After MARC-145 cells were incubated with different concentrations of Gel-FeS NPs (0‒340.0 µg/mL), the cells were collected at the indicated time points, and the content of Fe in the cells was detected by ICP-MS. **C** Analysis of intracellular ferrous iron release from Gel-FeS NPs by the ferrous ion colorimetric kit after incubating MARC-145 cells with different concentrations of Gel-FeS NPs (0‒340.0 µg/mL) and collecting the cells at the indicated time points. Control group: cells treated with the DMEM (2% FBS). Error bars represent the standard deviation from three repeated experiments

MARC-145 cells, an African green monkey kidney cell line, are highly sensitive to PRRSV, and are often used to isolate PRRSV for diagnostics, research, and vaccine production, leading to their frequent use in in vitro studies on PRRSV [39–41]. In order to study the antiviral activity of Gel-FeS NPs against PRRSV and define the experimental conditions, we first measured the biocompatibility of Gel-FeS NPs in MARC-145 cells, the host cell of PRRSV. After incubation with Gel-FeS NPs for 12, 24, 36, and 48 h, the viability of MARC-145 cells was measured by MTT assay. In Fig. 3, Gel-FeS NPs showed negligible cytotoxicity at the concentration below 430.0 µg/mL.

According to the TGA results, the mass fraction of Fe in Gel-FeS NPs is 17.8%, and 430.0 µg/mL of Gel-FeS NPs was estimated to contain 76.5 µg/mL iron element. The contents of internalized Gel-FeS in MARC-145 cells were evaluated by inductively coupled plasma-mass spectrometry (ICP-MS). As shown in Fig. 3B, the intracellular Fe content was about 4 pg/cell in the cells without any treatment, in contrast to 16 pg/cell in the cells incubated with 340.0 µg/mL Gel-FeS NPs, demonstrating that Gel-FeS NPs could enter the cells. Furthermore, we tested the intracellular release of Fe^2+^ by using the ferrous ion colorimetric kit, and the results are shown in Fig. 3C. After incubation with 340.0 µg/mL Gel-FeS NPs for 20 h, the intracellular release of ferrous ions was 4.1 pg/cell, which was higher than control group without any treatment (1.7 pg/cell), indicating the successful entry of Gel-FeS NPs into cells and the release of some ferrous ions from Gel-FeS NPs. According to the calculation, the Fe concentration used in these experiments ranged from 15.1–60.5 μg/mL, corresponding to the concentration range of Gel-FeS NPs (85.0–340.0 μg/mL), which is in the similar concentration range as previously reported by Dang et al. (0–48 μg/mL bio-assembled FeS nanoparticles for efficient cancer therapy) [42] and Song et al., who found Fe(II) nanoparticles did not affect the production of archaea at 0–300 μg/mL [43].

### Gel-FeS NPs exhibit inhibitory effect on PRRSV proliferation

**Fig. 4:**
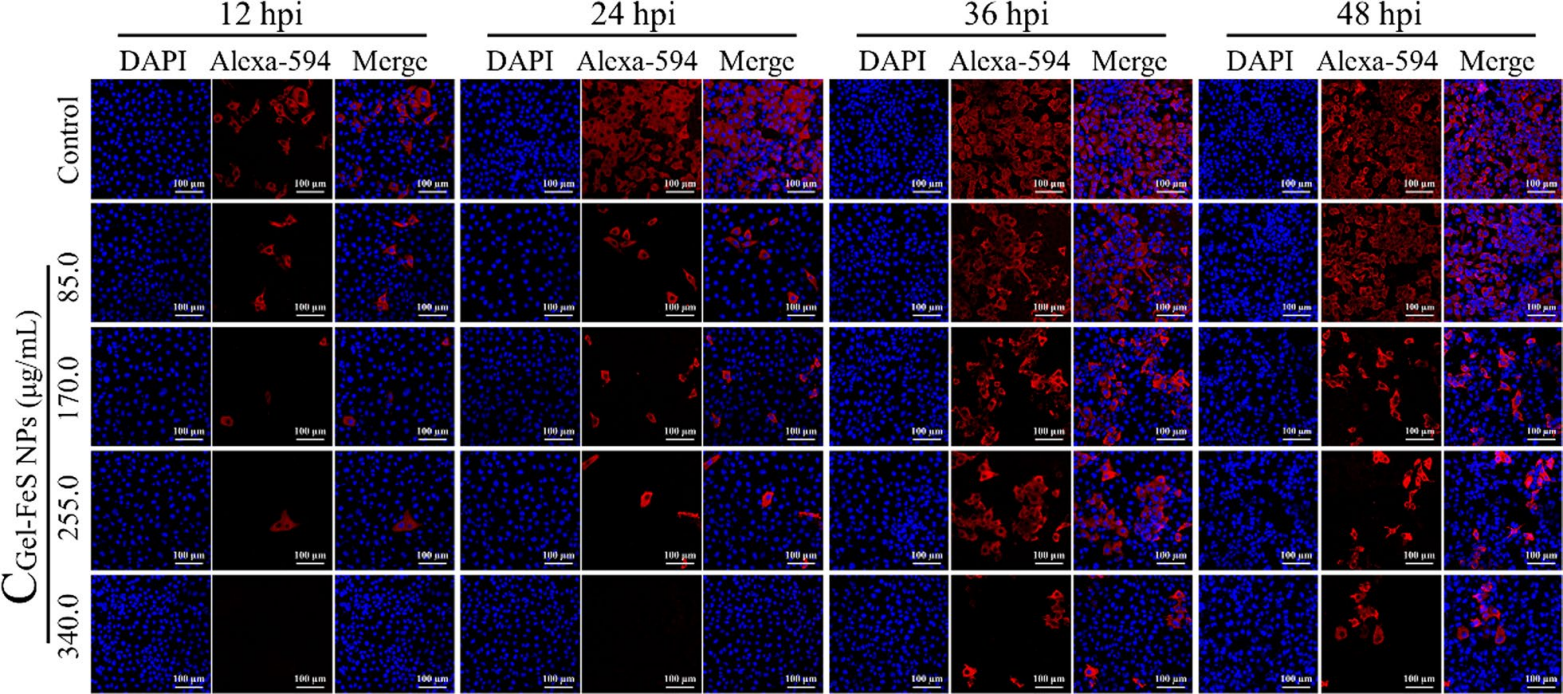
Gel-FeS NPs inhibited PRRSV infection in a time- and dose-dependent manner as indicated by indirect immunofluorescence assay. Immunofluorescence images of PRRSV-infected MARC-145 cells treated with different concentrations of Gel-FeS NPs (0‒340.0 µg/mL). The nucleus was stained with DAPI, and the viral protein was labeled with fluorescent dye Alexa Fluor® 594. Control group: the MARC-145 cells were not treated with nanomaterials, but inoculated with PRRSV. Scale bar: 100 μm

Based on the above cytotoxicity test results and the determined concentration range, we studied the antiviral activity of Gel-FeS NPs against PRRSV based on indirect immunofluorescence assay (IFA). In Fig. 4**,** compared with the control, the Gel-FeS NPs treated samples showed a significant time- and dose-dependent decline in the red fluorescence signal of Alexa Fluor® 594 labeled PRRSV N protein in the cytoplasm of MARC-145 cells, in contrast to no notable difference in the nuclei stained blue with DAPI, reflecting the good antiviral activity of Gel-FeS NPs.

**Fig. 5:**
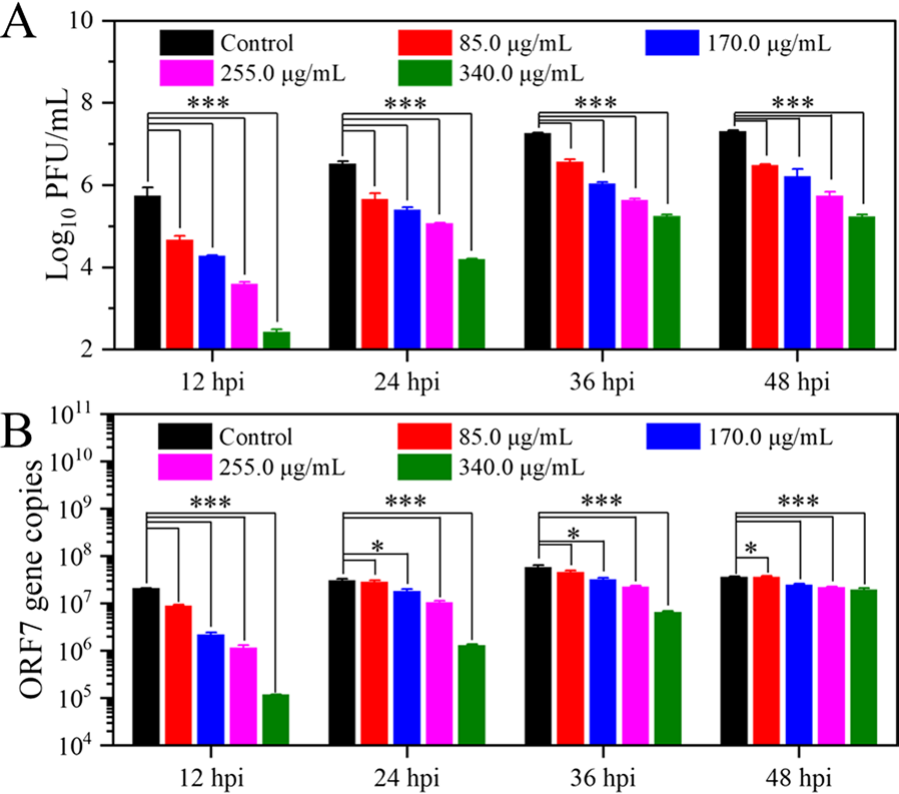
Quantitative analysis of inhibitory effects of Gel-FeS NPs on PRRSV infection. **A** Titers of PRRSV treated with different concentrations of Gel-FeS NPs (0‒340.0 µg/mL) detected by plaque reduction assay. **B** The content of ORF7 gene in PRRSV genome treated with different concentrations of Gel-FeS NPs (0‒340.0 µg/mL) was detected by RT-qPCR. Error bars represent the standard deviation from three repeated experiments. Control group: the MARC-145 cells were not treated with nanomaterials, but inoculated with PRRSV. The mean value was calculated by the t test (mean ± SD, *n* = 3). *p < 0.05, **p < 0.01, ***p < 0.001 indicate significant differences compared with the indicated group

Furthermore, the viral infectivity of PRRSV was determined by plaque reduction assay. In Fig. 5A, the titers of PRRSV treated by Gel-FeS NPs were seen to decrease significantly (p < 0.001) in a dose-dependent manner relative to the control group, resulting in ~ 10^3^-fold reduction in PRRSV titers. Next, we detected the RNA of PRRSV, which contains a single-stranded, positive-sense RNA genome with at least 10 open reading frames (ORFs), including ORF1a, ORF1b, ORF2a, ORF2b, ORF3, ORF4, ORF5, ORF5a, ORF6, and ORF7 [2, 44, 45]. Accordingly, the content of cellular ORF7 gene is proportional to the viral infection intensity and the number of infected cells. After incubation with Gel-FeS NPs, the cellular RNA of PRRSV infected cells was extracted and the ORF7 gene was detected by real-time quantitative reverse transcription polymerase chain reaction (RT-qPCR) to verify the inhibition of Gel-FeS NPs on virus proliferation. In Fig. 5B, the RT-qPCR results also demonstrated the excellent antiviral effect of Gel-FeS NPs on PRRSV proliferation.

### Gel-FeS NPs inhibit PRRSV proliferation in a multi-stage manner

**Fig. 6:**
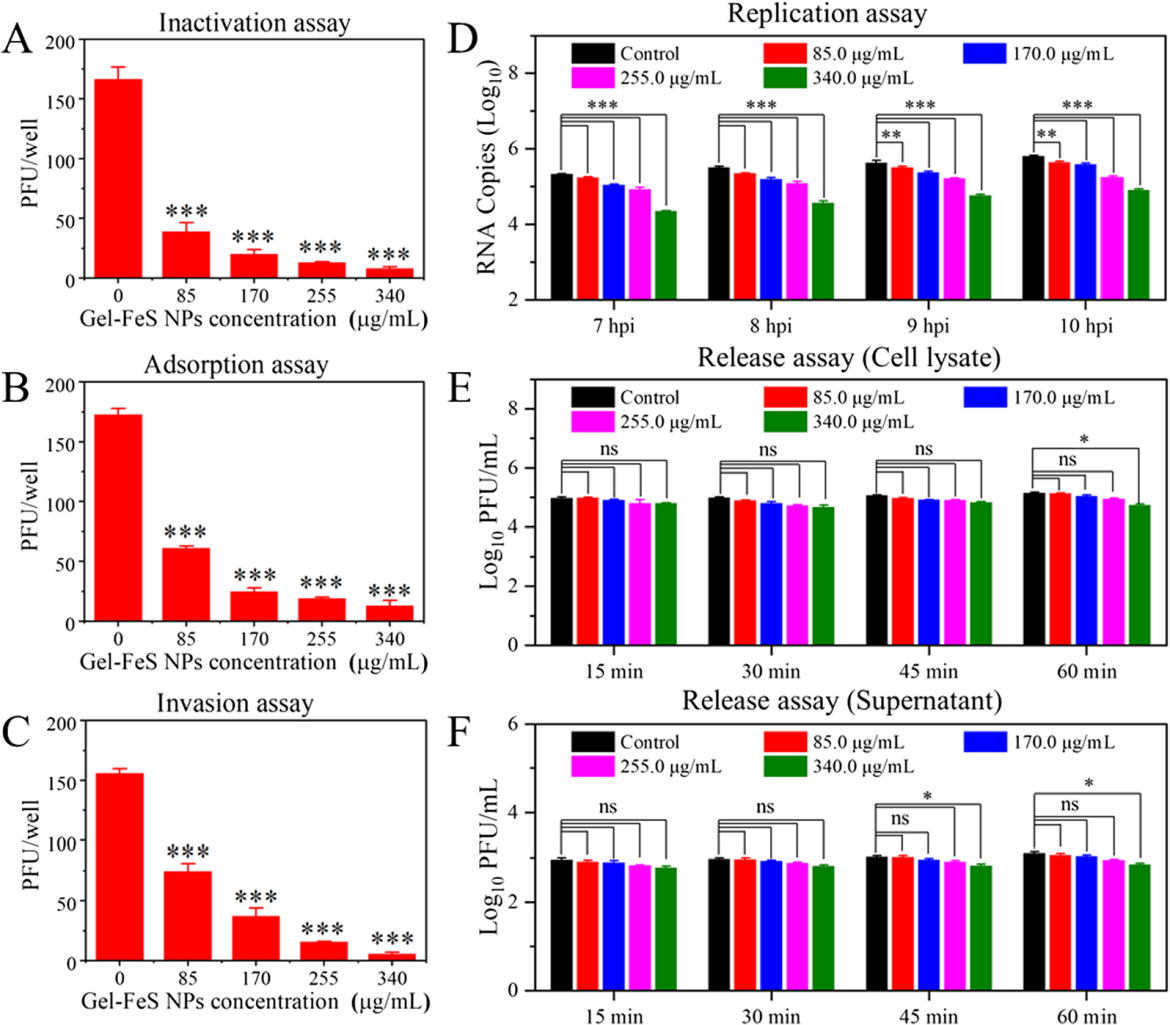
Multiple-stage effect of Gel-FeS NPs on PRRSV proliferation. **A** Inactivation effect of Gel-FeS NPs on PRRSV. The effect of Gel-FeS NPs on the PRRSV infectivity of MARC-145 cells in the infection process of **B** adsorption, **C** invasion, **D** replication, and **E**, **F** release (intracellular and supernatant samples, respectively). Error bars represent the standard deviation from three repeated experiments. Control group: the MARC-145 cells were not treated with nanomaterials, but inoculated with PRRSV. The mean value was calculated by the t test (mean ± SD, *n* = 3). *p < 0.05, **p < 0.01, ***p < 0.001 indicate significant differences compared with the indicated group

The effect of Gel-FeS NPs on the virus life cycle was investigated by analyzing their ability to directly inactivate PRRSV. In Fig. 6A, inactivation assay unveiled that the PRRSV content exhibited an extremely significant (p < 0.001) dose-dependent decrease, indicating that Gel-FeS NPs possess virucidal activity in vitro.

PRRSV infection involves a series of stages, and each stage may be a potential target of antiviral drugs. According to the reported life cycle characteristics of PRRSV and classical experimental methods [46], the effect of Gel-FeS NPs on each stage of PRRSV proliferation was explored, and the number of plaques can directly reflect their effect on the virus proliferation. In Fig. 6B, C, the PRRSV content showed ~ tenfold decrease in MARC-145 cells treated with Gel-FeS NPs during the two stages of adsorption and invasion, implying that Gel-FeS NPs could inhibit PRRSV adsorption and invasion to MARC-145 cells.

Following invasion by receptor-mediated endocytosis and disassembly, replicase polyproteins were produced under the guide of PRRSV genome positive strand RNA. This process is isolated as a PRRSV genome replication process, so the level of negative-sense RNA of PRRSV genome was quantified to assess the antiviral effect of Gel-FeS NPs on PRRSV replication by RT-qPCR. In Fig. 6D, the level of PRRSV negative-sense RNA decreased slightly after treatment with 340.0 μg/mL Gel-FeS NPs, suggesting that Gel-FeS NPs had a moderate inhibitory effect on PRRSV replication. In Fig. 6E, F, the release experimental results indicated the Gel-FeS NPs had no influence on PRRSV release, with no significant change observed in the virus content in either intracellular or supernatant samples. In general, Gel-FeS NPs could inhibit PRRSV adsorption, invasion, and replication in MARC-145 cells, but not its release in the cells.

### Inhibitory effect of ferrous ion on PRRSV proliferation

In order to further understand the inhibition mechanism of Gel-FeS NPs on PRRSV proliferation, we investigated the inhibitory effect of the individual synthetic raw materials (FeSO_4_, Na_2_S, and gelatin) on PRRSV proliferation. In Additional file 1: Fig. S5A and B, the cell relative viability was seen to be about 75% at 60.0 µg/mL Fe^2+^ concentration, in contrast to great reduction after incubation with 80.0 µg/mL of ferrous ions and S^2−^, but with little cytotoxicity at 40.0 µg/mL concentration. Meanwhile, gelatin exhibited good biocompatibility at high concentrations (0–340.0 µg/mL) (Additional file 1: Fig. S5C). Therefore, 40.0 µg/mL of Fe^2+^ and S^2−^ was selected to explore their antiviral activity by IFA, and the same concentration (255.0 µg/mL) as Gel-FeS NPs was used to explore the antiviral activity of gelatin. In Additional file 1: Fig. S5D, compared with the control cells, the cells treated by FeSO_4_ showed significant reduction in Alexa Fluor® 594 labeled PRRSV N protein, in contrast to no visible differences in the cells treated by Na_2_S and gelatin, indicating that the FeSO_4_ in the synthetic raw materials of Gel-FeS NPs had antiviral activity against PRRSV. This result confirmed our hypothesis that ferrous ion has antiviral activity, indicating the Fe(II) in Gel-FeS NPs plays a dominant role in inhibiting PRRSV proliferation.

The conclusion that Fe(II) inhibits virus proliferation can be further confirmed by the test results of CMC-FeS NPs and Gelatin NPs. In Additional file 1: Fig. S6A and B, CMC-FeS NPs showed similar cytotoxicity to Gel-FeS NPs, while Gelatin NPs showed better biocompatibility. In Additional file 1: Fig. S6C, the IFA results showed that CMC-FeS NPs had a certain antiviral effect and Gelatin NPs had no obvious antiviral effect at the same concentration of Gel-FeS NPs. These results further confirmed that Fe (II) plays a key role in inhibiting PRRSV proliferation.

## Discussion

Iron is an essential element for many organisms in maintaining the basic cellular physiological activities (such as oxygen transport, DNA replication, metabolic processes, etc.), and many proteins with crucial roles in cellular physiology require iron to function [37]. A series of diseases are associated with iron deficiency when iron-requiring enzymes become ineffective, such as many HIV-positive patients, who suffer from iron deficiency, which reduces their ability to resist viral infections, especially when the virus attacks immune cells [47]. Previous studies have shown that iron ions have antiviral activity and are essential for activating the host’s antiviral immune mechanism [48]. As an example, Fe (III) can effectively inhibit the replication of HSV-1 and bovine viral diarrhea virus (BVDV) [24], and it is also speculated to interact with viral RNA [49], which may hinder the replication of the viral genome. Extracellular iron, such as FAC, can also inhibit the infection of HIV-1 by suppressing the release of HIV-1 from lysosomes and block the replication of dengue virus by activating the activity of reactive oxygen species (ROS) in the intestinal epithelium of mosquitoes [25]. However, high concentrations of iron ions may produce strong cytotoxicity, and the survival rate of cells was shown to be less than 50% when cultured with 40 μg/mL ferrous ions. Therefore, how to improve the biocompatibility and antiviral effect of iron ion is of great significance for its application in antiviral field.

The present study found significant improvement in biocompatibility and antiviral effects by incorporating ferrous ions into Gel-FeS NPs. The ferrous sulfide nanoparticles synthesized here has an average size 47.3 nm, similar to the size of PRRS virions (50–74 nm) [44], which can inhibit virus proliferation through multiple stages. Our results indicated that Gel-FeS NPs could not only directly inactivate PRRSV in vitro, but also effectively inhibit PRRSV proliferation in the stages of adsorption and invasion, blocking the virus outside cells or from entering host cells, thus playing an important role in infection inhibition. As a highly biocompatible material, gelatin has been used in the research of drug delivery, wound adjuvant, vaccine adjuvant, and so on [49–52]. The targeting and antiviral effects of the gelatin-modified nanoparticles prepared here can be further improved by coating antiviral drugs or modified functional molecules on their surface in the future studies.

ROS, the by-products of cellular metabolism, are potential antiviral targets [53], and several viral infections were shown to cause an increase in intracellular ROS, mainly due to viral-induced imbalances in the antioxidant defense mechanisms of the cell, thereby activating certain host cellular pathways and promoting viral replication [54, 55]. Nano-sized iron (II) complex has been reported to possess excellent radical-scavenging activities and high antiviral activity against tobacco mosaic viruses (TMV) [56]. This suggests the possibility that Gel-FeS NPs might also inhibit viral infection by mediating the level of ROS induced by PRRSV infection, so we tested this possibility by the fluorescent probe DCFH-DA. The DCFH-DA results showed no significant difference between the control and Gel-FeS NPs-treated cells in the intensity of the green fluorescence signals (Additional file 1: Fig. S7), indicating that Gel-FeS NPs do not inhibit PRRSV proliferation by decreasing its ROS production in the host cell.

In this work, we found that the integration of ferrous ions into Gel-FeS NPs could significantly improve the biocompatibility and antiviral effects of ferrous ions-based NPs. They were demonstrated high antiviral activity against PRRSV proliferation in the stages of adsorption, invasion, and replication. Moreover, the Fe^2+^ released from degraded Gel-FeS NPs still displayed an antiviral effect, indicating the advantage of Gel-FeS NPs as an antiviral nanomaterial compared to other nanomaterials. This work highlighted the antiviral effect of Gel-FeS NPs and provided a new strategy for ferrous-based nanoparticles against PRRSV. However, despite their good antiviral effect in vitro, the in vivo and clinical applications of Gel-FeS NPs need more consideration, such as adjusting the synthesis and storage method according to the type of antiviral drugs (oral or injection).

## Conclusion

In this paper, we reported the virucidal and antiviral activity of Gel-FeS NPs for the first time. The Gel-FeS NPs with good dispersibility and biocompatibility were large-scale synthesized rapidly in a one-pot method, and they exhibited effective inhibition on PRRSV adsorption, invasion, and replication in MARC-145 cells. Moreover, the ferrous ions from degraded ferrous sulfide still displayed an antiviral effect, demonstrating the advantage of Gel-FeS NPs as an antiviral nanomaterial relative to other nanomaterials. This work highlighted the antiviral effect of Gel-FeS NPs and provided a new strategy for ferrous-based nanoparticles against PRRSV.

## Methods

### Preparation of FeS NPs

Stable Gel-FeS NPs were synthesized through a co-precipitation method based on the reaction of FeSO_4_ with Na_2_S in the presence of gelatin. Briefly, under continuous magnetic stirring and high-purity nitrogen flow conditions, 20.0 mL FeSO_4_ (0.020 mol/L) and 50.0 mL gelatin (1.0 g/L) solutions were mixed in a three-necked flasks, followed by stirring the mixture for 30 min to yield Fe^2+^-gelatin complexes, and adding 20.0 mL Na_2_S (0.020 mol/L) solution dropwise at an Fe-to-S molar ratio of 1:1 to produce FeS nanoparticles [31]. After centrifugation of the obtained solution at 10,000 rpm for 10 min, the supernatant was collected and directly vacuum freeze-dried to solid state for storage.

For synthesizing CMC-FeS NPs, CMC was used instead of gelatin, and the synthesis method is similar as described above.

### Cytotoxicity assay

MARC-145 cells were seeded in 96-well plates and cultured to approximately 80–90% confluence, followed by incubation with Gel-FeS NPs at different concentrations (0, 14.0, 28.0, 54.0, 108.0, 215.0, and 430.0 µg/mL). Meanwhile, cells treated with the DMEM (2% FBS) were used as the control. After incubation separately for 12, 24, 36 and 48 h, the standard MTT assay was used to evaluate the cell viability [57].

### Cell internalization experiment of Gel-FeS NPs

MARC-145 cells were seeded in 24-well plates and cultured to approximately 100% confluence, followed by incubation with Gel-FeS NPs at different concentrations (0, 170.0 and 340.0 µg/mL). Meanwhile, cells treated with the DMEM (2% FBS) were used as the control. After incubation separately for 0.5, 1.5, 3 and 5 h, the supernatant was removed, followed by 3 washes of the cells with PBS, and digesting the cells with 0.25% trypsin solution (WISENT, 325-043-EL) to a single cell. All trypsin digested cell samples were collected and nitrated with nitric acid (AR, Sinopharm Chemical Reagent) overnight. Iron standard (Aladdin, 7439-89-6) was purchased and diluted to the specified concentration (20–100 ppb), the samples were diluted with 1% dilute nitric acid for ICP-MS detection. The experiment was repeated three times in order to calculate the relative standard deviation.

### Intracellular ferrous ion release assay

MARC-145 cells were seeded in 24-well plates and cultured to approximately 100% confluence, followed by incubation with Gel-FeS NPs at different concentrations (0, 170.0 and 340.0 µg/mL). Meanwhile, cells treated with the DMEM (2% FBS) were used as the control. After incubation separately for 2, 6, 10 and 20 h, the supernatant was removed, followed by washing the cells 3 times with PBS, and digesting the cells with 0.25% trypsin solution (without EDTA and phenol red) (Solarbio, T1350) to a single cell. The content of intracellular ferrous ions was detected as instructed by the manufacturer (Elabscience, E-BC-K304-S).

### Antiviral assay

MARC-145 cells were incubated with Gel-FeS NPs at different concentrations (0, 85.0, 170.0, 255.0, and 340.0 µg/mL) for 2 h at 37 °C. Meanwhile, PRRSV was pretreated with Gel-FeS NPs at 4 °C for 1 h. Then, the cells were incubated with the pretreated PRRSV at the multiplicity of infection (MOI) of 1.0 for 1 h. Next, the inoculums were discarded, followed by two washes with DMEM, and incubation of the cells with Gel-FeS NPs at the corresponding concentration and 37 °C for 12, 24, 36 and 48 h, respectively. Finally, the cell samples were collected for IFA and plaque reduction assay [46].

### Indirect immunofluorescence assay

MARC-145 cells were cultured in 24-well plates and collected at indicated time points, followed by three washes with PBS, treating the cells with 4% paraformaldehyde for 15 min, and permeabilizing the cells with precooled methanol at − 20 ℃ for 15 min. Next, the cells were blocked by 5% (w/v) BSA for 45 min and then detected with a mouse monoclonal antibody (primary antibodies) against the PRRSV N protein and Alexa Fluor® 594-conjugated Donkey anti mouse lgG (secondary antibodies), Cell nucleus was stained by DAPI [58]. The fluorescence images were acquired with Laser confocal microscope LSM880 (ZEISS, Germany).

### Plaque reduction assay

Briefly, MARC-145 cells were seeded in 6-well plates and cultured until ~ 100 confluence, followed by adding the indicated samples to the plates by tenfold gradient dilution with DMEM (2% FBS) and incubation for 1.5 h. After discarding the supernatant, the cells were washed three times with DMEM to remove non-absorbed PRRS virions, followed by adding to each well 2 mL overlay medium (2 × DMEM:low melting point agarose 1.8% (w/w):FBS:penicillin–streptomycin = 48:48:3:1), and cooling for 15 min at 4 °C to coagulate the overlay medium. After incubation at 37 °C for 2 ~ 3 days, the cells were stained with 1.0 mL neutral red solution (0.50 mg/mL) for 1 h at 37 °C, followed by removing the supernatant and storing the plates at 4 °C overnight [59]. Finally, the number of plaques was counted and virus titers were calculated. All the virus titers were presented as plaque forming units (PFU/mL).

### Inactivation assay

The plaque assay was performed to investigate whether Gel-FeS NPs could directly inactivate PRRSV. Briefly, different contents of PRRSV (MOI = 1.0, 0.1, 0.01, 0.001, and 0.0001) were used for pre-experiments to find a suitable PRRSV concentration for plaque assay of the effect of Gel-FeS NPs on the inactivation of PRRSV [60]. PRRSV (MOI = 0.001) was incubated with Gel-FeS NPs at different concentrations (0, 85.0, 170.0, 255.0, and 340.0 µg/mL) for 1 h at 37 °C. The 6-well plates were pre-chilled separately at 4 °C for 30 min when cells reached 100% confluence, followed by adding the pretreated PRRSV to the 6-well plates. After incubation at 4 °C for 2 h, the cells were covered with overlay medium for the corresponding plaque assay.

### Adsorption assay

The MARC-145 cells cultured in 6-well plates were pre-chilled at 4 °C for 30 min, followed by incubation with the PRRSV (MOI = 0.001) and Gel-FeS NPs of different concentrations (0, 85.0, 170.0, 255.0, and 340.0 µg/mL) at 4 °C for 2 h [61]. Finally, the cells were washed three times with pre-cooled DMEM and covered with overlay medium for the corresponding plaque assay.

### Invasion assay

The MARC-145 cells were cultured in 6-well plates and pre-chilled at 4 °C for 30 min, followed by infection with PRRSV (MOI = 0.001) at 4 °C for 2 h, and three washes with pre-cooled DMEM. Next, the virus-containing medium was replaced by fresh medium containing Gel-FeS NPs of different concentrations (0, 85.0, 170.0, 255.0, and 340.0 µg/mL). After incubation at 37 °C for 3 h, the supernatant was discarded, and the cells were covered with overlay medium for the corresponding plaque assay [62].

### Replication assay

The MARC-145 cells in 24-well plates were infected with PRRSV (MOI = 1.0) at 37 °C for 1 h, followed by replacing the supernatant with DMEM (2% FBS) and culturing the cells for additional 6 h. After removing the supernatant, 24-well plates were supplemented with DMEM containing Gel-FeS NPs of different concentrations (0, 85.0, 170.0, 255.0, and 340.0 µg/mL). After incubation for another 7, 8, 9 and 10 h, the total RNA of the infected cells was extracted using the TRIzol reagent as instructed by the manufacturer. TAKARA Reverse Transcription Kit was used to reverse transcribe RNA to cDNA according to the manufacturer’s instructions with the primer (5′UF: GACGTATAGGTGTTGGCTC). PCR assay was performed using the primers (F: GCATTTGTATTGTCAGGAGC, R: AGCAGTGCAACTCCGGAAG) [47]. The abundance of PRRSV negative-sense RNA was quantified by RT-qPCR assay.

### Release assay

The MARC-145 cells were infected with PRRSV (MOI = 1.0) at 37 °C for 1 h, followed by discarding the supernatant and culturing the cells in DMEM (2% FBS) at 37 °C for another 18 h. After three washes with DMEM, 24-well plates were added with fresh DMEM containing different concentrations of Gel-FeS NPs (0, 85.0, 170.0, 255.0, and 340.0 µg/mL). After incubation for 15, 30, 45 and 60 min, the PRRSV supernatant samples were collected, followed by adding fresh DMEM and collecting the cell lysate by freezing and thawing three times [63]. Finally, a plaque assay was performed to determine the virus content of the corresponding samples.

## Supplementary Information

**Additional file 1.** Chemicals and Reagents, Viruses and Cell Culture, Preparation of gelatin nanoparticles, Characterization of Gel-FeS NPs, Control experiment for the antiviral effect of raw materials and other nanoparticles, Measurement of the production of reactive oxygen species. **Figure S1.** The morphological picture and UV-Vis spectra of Gel-FeS NPs. **Figure S2.** Pictures of Gel-FeS NPs dispersed in different solvents. **Figure S3.** TEM images of CMC-FeS NPs and Gelatin NPs. **Figure S4.** Dynamic light scattering distribution images of CMC-FeS NPs and Gelatin NPs. **Figure S5.** Cytotoxicity and antiviral activity assay of of different concentrations of Fe2+, S2- and gelatin. **Figure S6.** Cytotoxicity and antiviral activity of FeS NPs and Gelatin NPs. **Figure S7.** Effect of Gel-FeS NPs on PRRSV-induced ROS production.

## Data Availability

Not applicable.

## Abbreviations

PRRSV: Porcine reproductive and respiratory syndrome virus
FTIR: Fourier transmission infrared spectroscopy
TEM: Transmission electron microscope
XRD: X Ray diffraction
XPS: X-ray Photoelectron Spectroscopy
MTT: 3-(4,5-Dimethylthiazol-2-yl)-2,5-diphenyltetrazolium bromide
IFA: Indirect immunofluorescence assay
RT-qPCR: Real-time quantitative reverse transcription polymerase chain reaction
