# Supplementary material for: Inhibitory effect and mechanism of gelatin stabilized ferrous sulfide nanoparticles on porcine reproductive and respiratory syndrome virus
Source: J Nanobiotechnology. 2022 Feb 5;20:70. doi: 10.1186/s12951-022-01281-4 (PMC8817501; doi:10.1186/s12951-022-01281-4)
Supplement: Supplementary file 1 — Additional file 1. Chemicals and Reagents, Viruses and Cell Culture, Preparation of gelatin nanoparticles, Characterization of Gel-FeS NPs, Control experiment for the antiviral effect of raw materials and other nanoparticles, Measurement of the production of reactive oxygen species. Figure S1. The morphological picture and UV-Vis spectra of Gel-FeS NPs. Figure S2. Pictures of Gel-FeS NPs dispersed in different solvents. Figure S3. TEM images of CMC-FeS NPs and Gelatin NPs. Figure S4. Dynamic light scattering distribution images of CMC-FeS NPs and Gelatin NPs. Figure S5. Cytotoxicity and antiviral activity assay of of different concentrations of Fe2+, S2- and gelatin. Figure S6. Cytotoxicity and antiviral activity of FeS NPs and Gelatin NPs. Figure S7. Effect of Gel-FeS NPs on PRRSV-induced ROS production. [file 12951_2022_1281_MOESM1_ESM.docx]

**Additional materials**

**Author information**

Ting Tong: tongting@webmail.hzau.edu.cn

Shuangfei Deng: 2776318316@qq.com

Xiaotong Zhang: 18827053900@163.com

Liurong Fang: fanglr@mail.hzau.edu.cn

Jiangong Liang: liangjg@mail.hzau.edu.cn

Shaobo Xiao: [vet@mail.hzau.edu.cn](mailto:vet@mail.hzau.edu.cn)

**Experimental methods**

Chemicals and Reagents

FeSO_4_·7H_2_O (AR, ≥ 99%), Na_2_SO_4_·9H_2_O (AR, ≥ 98%), Acetone (AR, ≥ 99.5%), and dimethyl sulfoxide (DMSO, CP, ≥ 98%) were purchased from Sinopharm Chemical Reagent Co., Ltd., China; Glutaraldehyde (50%), gelatin and carboxymethyl cellulose (CMC) from Shanghai Aladdin Chemistry Co. Ltd., China. All the other experimental chemicals of analytical grade were obtained from Sigma-Aldrich China, Inc., (Shanghai, China), and used directly without further purification. FBS (BR) was purchased from Gibco (NY, USA), low melting point agarose (BR) from Promega Corporation (Madison, USA). Deionized water was obtained from a Millipore water purification system (Milli-Q, Millipore, 18.25 MΩ).

Viruses and Cell Culture

Monkey kidney (MARC-145) cells were purchased separately from the American Type Culture Collection (ATCC) and the China Center for Type Culture Collection (CCTCC). Cells were cultured in Dulbecco’s modified Eagle’s medium (DMEM) supplemented with 10% fetal bovine serum (FBS) and 1% penicillin/streptomycin at 37 °С in a humidified CO_2_ incubator. The PRRSV strain WUH3 (GenBank Accession No. HM853673) (isolated at the end of 2006 in China from the pig brains with the syndrome of “high fever”) was transfected into MARC-145 cells as previously reported [1, 2].

Preparation of gelatin nanoparticles

The preparation of gelatin nanoparticles followed a previously reported method [3] with partial modifications. Briefly, gelatin (1.25 g) was dissolved in 25 mL of distilled water under constant heating and stirring, followed by adding 25 mL of acetone to the solution successively and dilution with water until the gelatin concentration of 5 g/L. After adjusting the solution pH to 8.0 with 1 M sodium hydroxide solution, a certain amount of acetone was added dropwise until the solution became milky white, followed by adding 5.625 mL the glutaraldehyde (50%) and stirring the mixture in a 40 ℃ water bath for 16 h. After filtering the mixture through a 0.22 μm filter membrane and freeze-drying to remove solvent acetone and water, the resulting solid gelatin nanoparticles were dissolved in water to prepare a 10 mg/mL gelatin nanoparticle aqueous solution for subsequent experiments.

Characterization of Gel-FeS NPs

UV-Vis absorption spectra were recorded from 200 nm to 500 nm on a Shimadzu UV-2450 spectrometer (Shimadzu, Japan) equipped with a 10 mm quartz cell. The FTIR spectra were recorded with a Thermo Fisher Nicolet Avatar-330 infrared spectrometer (Thermo, USA) and scanned between 4000 and 500 cm^−1^. TEM images were obtained by a H-7650 transmission electron microscope (HITACHI, Japan). HR-TEM images were obtained by a JEM-2100F transmission electron microscope (JEOL Japan). The X-ray diffraction (XRD) was conducted with a D8 Advance X-ray Diffractometer (BRUKER, Germany) with Cu Kα radiation at 40 kV and 10° min^−1^. XPS scanning was performed on a Thermo Fisher ESCALAB Xi X-ray photoelectron spectrometer (Thermo, USA) to obtain the surface elemental composition and chemical state of the sample. Zeta potential and hydrodynamic diameters were obtained by using a Zetasizer Nano ZS90 dynamic light scattering (DLS) system (Malvern, England). Thermogravimetric analysis (TGA) was performed with a DSC200PC analyzer (NETZSCH, Germany). The results of cytotoxicity assay and ferrous ion colorimetry were obtained using an Envision Microplate Reader (PE, USA). The gene content was detected by Real time fluorescence quantitative PCR (RT-qPCR) instrument Quant Studio 7 (ABI, USA). The indirect immunofluorescence results were obtained by Laser confocal microscope LSM880 (ZEISS, Germany) or inverted fluorescence microscope Ti-U (Nikon, Japan). The iron content was determined using an inductively coupled plasma mass (ICP-MS) 7900 spectrometer (Agilent, USA).

Control experiment for the antiviral effect of raw materials and other nanoparticles

For measuring the effects of individual raw materials on cell relative viability, MARC-145 cells were seeded in 96-well plates to a confluence of approximately 80-90%, followed by incubation separately with gelatin, ferrous ammonium sulfate, Na_2_S, Gel-FeS NPs, CMC- FeS NPs, and gelatin NPs at different concentrations in DMEM supplemented with 2% FBS for 36 h. After replacing the supernatant with 100 µL of fresh DMEM (2% FBS), each well was supplemented with 20 µL of MTT (3- [4,5- dimethylthiazol-2-thiazolyl]-2,5-diphenyl tetrazolium bromide, Sigma) solution (5.0 mg/mL). After incubation for 4 h, the supernatant was removed, and the formazan crystals were dissolved in 150 µL per well of dimethyl sulfoxide (DMSO). After shaking on a rocking shaker for 10 min at 150 rpm, the OD values at 630 nm were measured for estimating the percentage of cell relative viability using a microplate reader.

For measuring the antiviral effect of individual raw materials, MARC-145 cells were treated separately with gelatin, ferrous ammonium sulfate, Na_2_S, Gel-FeS NPs, CMC- FeS NPs, and gelatin NPs for 2 h at 37 °C. Meanwhile, PRRSV was preincubated with gelatin, ferrous ammonium sulfate, Na_2_S, Gel-FeS NPs, CMC- FeS NPs, and gelatin NPs at the corresponding concentrations for 1 h at 4 °C. Then, the supernatant of MARC-145 cells was substituted with the pretreated PRRSV at the multiplicity of infection (MOI) of 1.0 for 1 h at 37 °C. Next, the supernatant was discarded, followed by two washes with DMEM, and incubation separately with gelatin, ferrous ammonium sulfate, Na_2_S, Gel-FeS NPs, CMC- FeS NPs, and gelatin NPs at the corresponding concentrations for 36 h. Finally, their individual antiviral effect on PRRSV infection was evaluated by indirect immunofluorescence assay.

Measurement of the production of reactive oxygen species (ROS)

The ROS levels were measured using the ROS kit (Beyotime, S0033S) as instructed by the manufacturer. Briefly, the MARC-145 cells were incubated with different concentrations of Gel-FeS NPs (0-340.0 μg/mL) and PRRSV (1.0 MOI) as described in Antiviral assay. At 36 hours post infection (hpi), the cells were incubated with dichloro-dihydro-fluorescein diacetate (DCFH-DA), which was diluted to the final concentration of 5.0 μmol/L. After incubation of 0.5 h, the supernatant was substituted with PBS. After removing the staining solution and washing twice with PBS, an inverted fluorescence microscope was used to evaluate the relative ROS level by observing the fluorescence images. An argon laser with a wavelength of 488 nm was used for excitation, and the fluorescent dichlorofluorescein (DCF) was analyzed at an emission wavelength of 525 nm.

**Supplementary Figure S1**

**
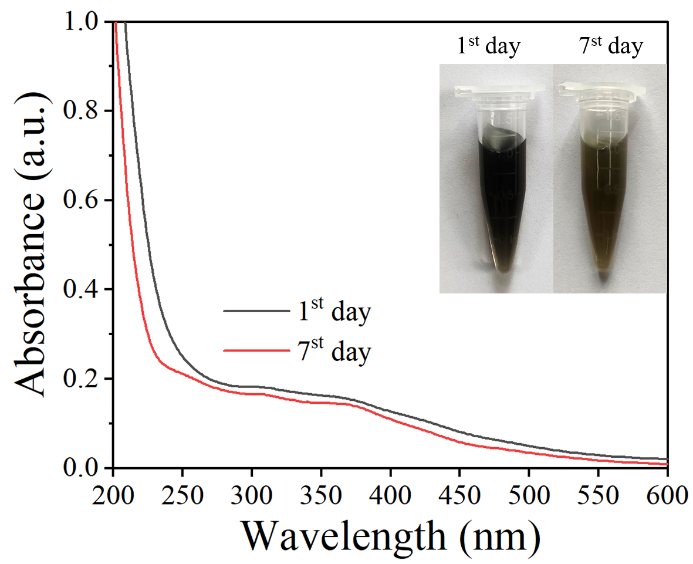
**

**Fig. S1.** The morphological picture and UV-Vis spectra of Gel-FeS NPs

**Supplementary Figure S2**


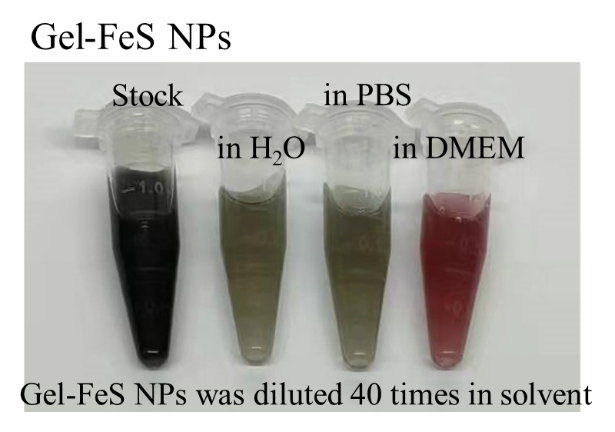


**Fig. S2.** Pictures of Gel-FeS NPs dispersed in different solvents.

**Supplementary Figure S3**


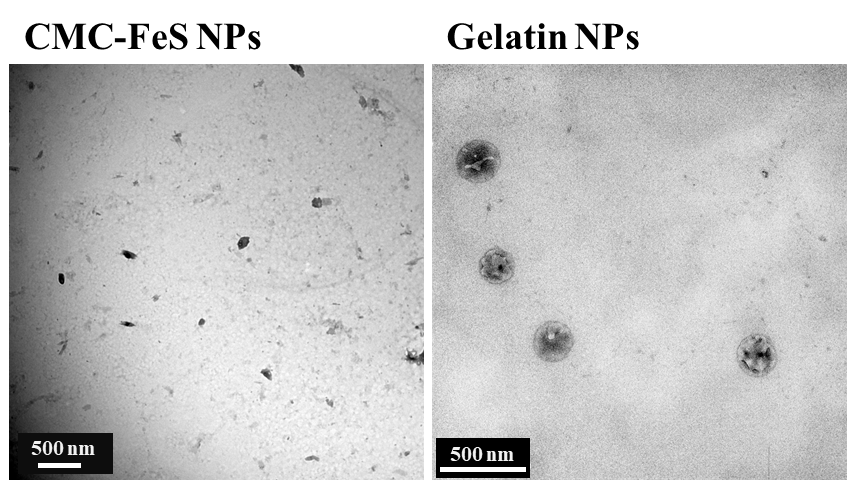


**Fig. S3.** TEM images of CMC-FeS NPs and Gelatin NPs.

**Supplementary Figure S4**


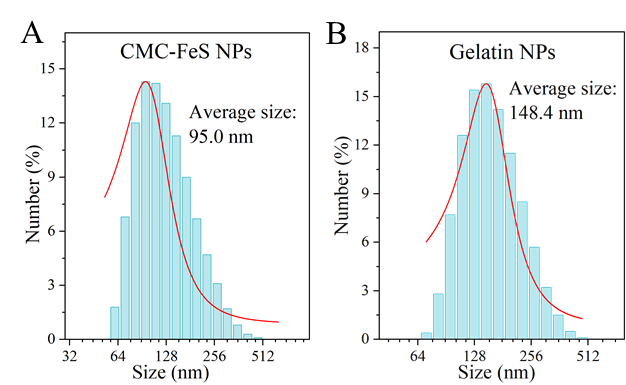


**Fig. S4.** Dynamic light scattering distribution images of CMC-FeS NPs and Gelatin NPs.

**Supplementary Figure S5**


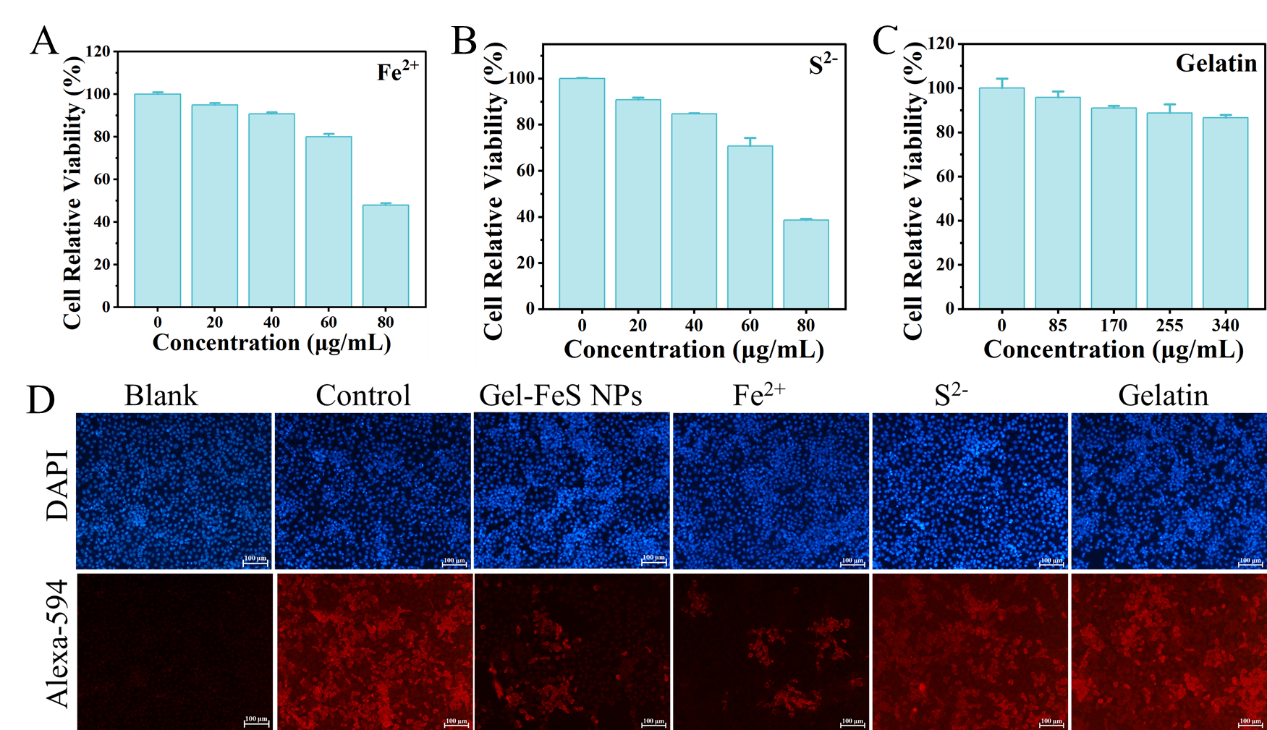


**Fig. S5**. Cytotoxicity and antiviral activity assay. Cytotoxicity of different concentrations of (A) Fe^2+^ (0-80.0 µg/mL), (B) S^2-^ (0-80.0 µg/mL) and (C) gelatin (0-340.0 µg/mL) on MARC-145 cells by MTT assay. (D) Immunofluorescence images of PRRSV-infected MARC-145 cells treated separately with Gel-FeS NPs (255.0 µg/mL), Fe^2+^ (40.0 µg/mL), S^2-^ (40.0 µg/mL), and Gelatin (255.0 µg/mL); the nucleus was stained by DAPI, and the N protein of PRRSV was labeled by Alexa Fluor® 594. Blank group: the MARC-145 cells were not treated with nanomaterials or PRRSV. Control group: the MARC-145 cells were not treated with nanomaterials, but inoculated with PRRSV. Scale bar = 100 µm.

**Supplementary Figure S6**


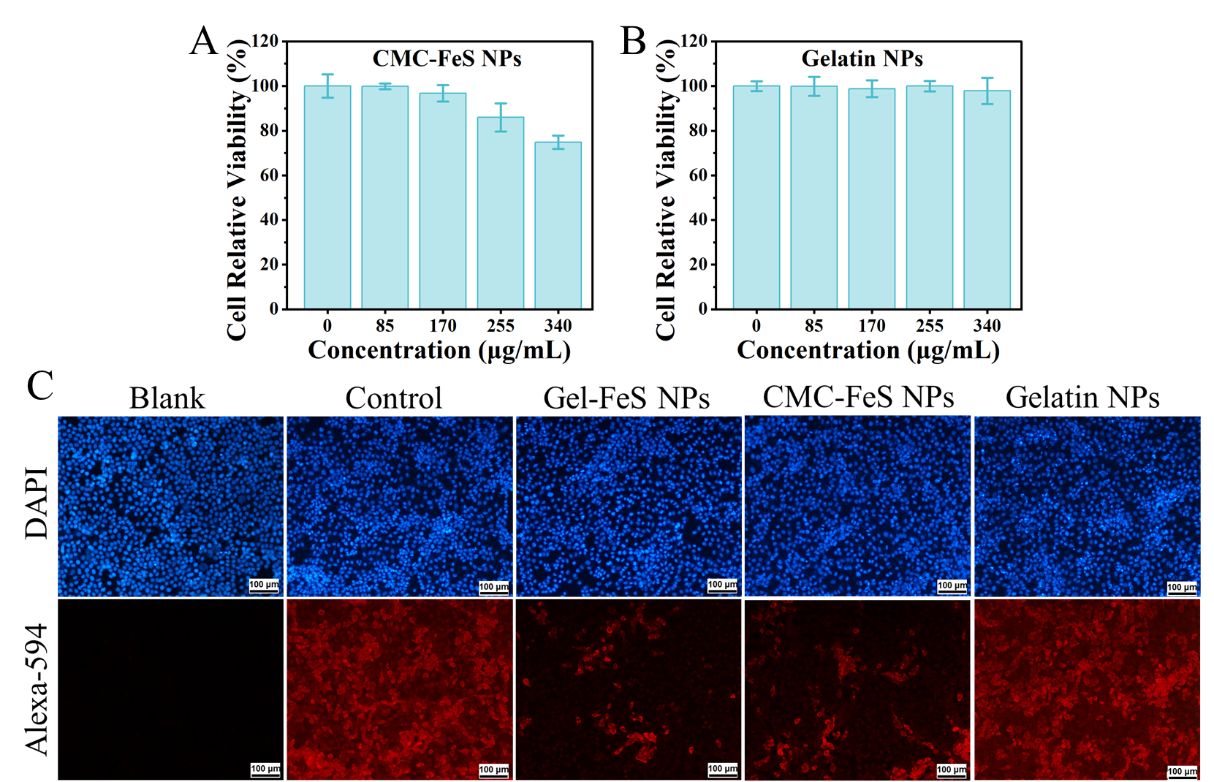


**Fig. S6**. Cytotoxicity and antiviral activity of FeS NPs and Gelatin NPs. Cytotoxicity of different concentration of (A) CMC-FeS NPs (0-340.0 µg/mL) and (B) gelatin NPs (0-340.0 µg/mL) on MARC-145 cells detected by MTT assay. (C) Immunofluorescence images of PRRSV-infected MARC-145 cells treated with Gel-FeS NPs, CMC-FeS NPs and gelatin NPs at the same concentrations (255.0 µg/mL). The nucleus was stained by DAPI, and the N protein of PRRSV was labeled red by Alexa Fluor® 594. Blank group: the MARC-145 cells were not treated with nanomaterials or PRRSV. Control group: the MARC-145 cells were not treated with nanomaterials, but inoculated with PRRSV. Scale bar = 100 µm.

**Supplementary Figure S7**


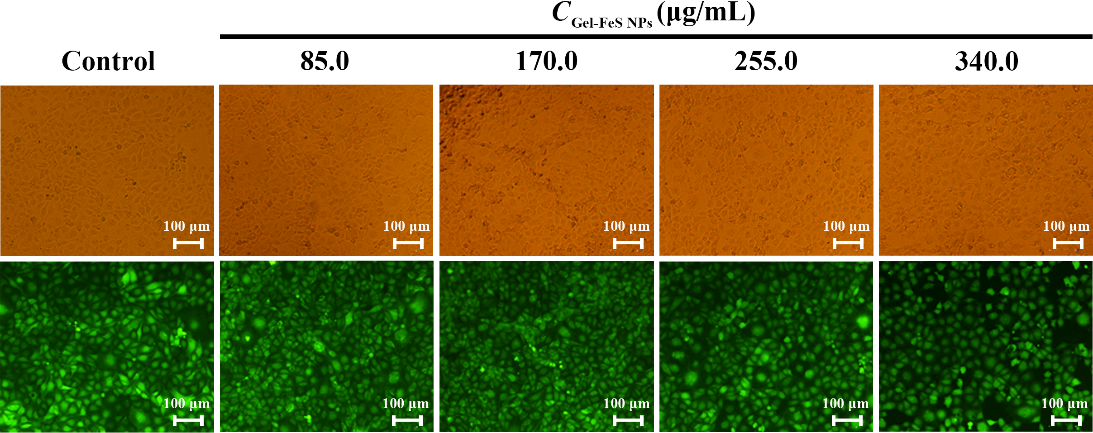


**Fig. S7** Effect of Gel-FeS NPs on PRRSV-induced ROS production as evaluated by DCFH-DA. Control group: the MARC-145 cells were not treated with nanomaterials, but inoculated with PRRSV.

**References**

1. Wang D, Cao L, Xu Z, Fang LR, Zhong Y, Chen QG, Luo R, Chen HC, Li K, Xiao SB. MiR-125b Reduces Porcine reproductive and respiratory syndrome virus replication by negatively regulating the nf-kappa b pathway. PLoS One. 2013;8(2):e55838.
2. Li B, Xiao SB, Wang YW, Xu SS, Jiang YB, Chen HC, Fang LR. Immunogenicity of the highly pathogenic porcine reproductive and respiratory syndrome virus GP5 protein encoded by a synthetic ORF5 gene. Vaccine. 2009;27(13):1957-1963.
3. Tan H, Sun GQ, Lin W, Mu CD, Ngai T. Gelatin particle-stabilized high internal phase emulsions as nutraceutical containers. ACS Appl Mater Interfaces. 2014;6(16):13977-13984.
